# Effect of addition of nano-hydroxyapatite on physico-chemical and antibiofilm properties of calcium silicate cements

**DOI:** 10.1590/1678-775720150422

**Published:** 2016

**Authors:** Juliane Maria GUERREIRO-TANOMARU, Fernando Antonio VÁZQUEZ-GARCÍA, Roberta BOSSO-MARTELO, Maria Inês Basso BERNARDI, Gisele FARIA, Mario TANOMARU

**Affiliations:** 1 - Universidade Estadual Paulista, Faculdade de Odontologia de Araraquara, Departamento de Odontologia Restauradora, Araraquara, SP, Brasil.; 2 - Universidade de São Paulo, Instituto de Física, São Carlos, SP, Brasil.

**Keywords:** Physicochemical analysis, Biofilms, Enterococcus faecalis, Zirconium, Nanotechnology

## Abstract

**Objective:**

Mineral Trioxide Aggregate (MTA) is a calcium silicate cement composed of Portland cement (PC) and bismuth oxide. Hydroxyapatite has been incorporated to enhance mechanical and biological properties of dental materials. This study evaluated physicochemical and mechanical properties and antibiofilm activity of MTA and PC associated with zirconium oxide (ZrO_2_) and hydroxyapatite nanoparticles (HAn).

**Material and Methods:**

White MTA (Angelus, Brazil); PC (70%)+ZrO_2_ (30%); PC (60%)+ZrO_2_ (30%)+HAn (10%); PC (50%)+ZrO_2_ (30%)+HAn (20%) were evaluated. The pH was assessed by a digital pH-meter and solubility by mass loss. Setting time was evaluated by using Gilmore needles. Compressive strength was analyzed by mechanical test. Samples were radiographed alongside an aluminum step wedge to evaluate radiopacity. For the antibiofilm evaluation, materials were placed in direct contact with *E. faecalis* biofilm induced on dentine blocks. The number of colony-forming units (CFU mL-1) in the remaining biolfilm was evaluated. The results were submitted to ANOVA and the Tukey test, with 5% significance.

**Results:**

There was no difference in pH levels of PC+ZrO_2_, PC+ZrO_2_+HAn (10%) and PC+ZrO_2_+HAn (20%) (p>0.05) and these cements presented higher pH levels than MTA (p<0.05). The highest solubility was observed in PC+ZrO_2_+HAn (10%) and PC+ZrO_2_+HAn (20%) (p<0.05). MTA had the shortest initial setting time (p<0.05). All the materials showed radiopacity higher than 3 mmAl. PC+ZrO_2_ and MTA had the highest compressive strength (p<0.05). Materials did not completely neutralize the bacterial biofilm, but the association with HAn provided greater bacterial reduction than MTA and PC+ZrO_2_ (p<0.05) after the post-manipulation period of 2 days.

**Conclusions:**

The addition of HAn to PC associated with ZrO_2_ harmed the compressive strength and solubility. On the other hand, HAn did not change the pH and the initial setting time, but improved the radiopacity (HAn 10%), the final setting time and the *E. faecalis* antibiofilm activity of the cement.

## INTRODUCTION

Mineral Trioxide Aggregate (MTA) is a calcium silicate cement composed of Portland cement (PC) and bismuth oxide (Bi_2_O_3_)^24^. MTA shows proper physicochemical properties, biocompatibility, bioactivity and antimicrobial activity^27^ ^,^ ^30^.

The Bi_2_O_3_ replacement in MTA composition has been suggested, since it interferes in the solubility, pH, and setting time^27^. The PC association with 30% zirconium oxide (ZrO_2_) exhibited radiopacity, compressive strength, setting time, water uptake, solubility and sorption comparable to ProRoot MTA^16^. Bioactive potential have been observed for the PC/ZrO_2_ association^15^.

Hydroxyapatite have been incorporated into root canal sealers^14^, retrofilling materials^4^, dental composites^2^ and adhesive resin systems^28^ to enhance the mechanical, physicochemical and biological properties of materials. Nanohydroxyapatite (HAn) is considered a biocompatible and bioactive material^17^ and presents osteoconductive property^29^. Dasgupta, et al.^17^ (2013) evaluated the properties presented by micro and nanoparticles of hydroxyapatite (HAn), and observed better physical, mechanical and biological properties for the material with particle sizes of 168 nm. Therefore, the addition of HAn could be an option for improving the properties of the calcium silicate-based cements.

The aim of this study was to evaluate physicochemical, mechanical and antibiofilm properties against *E faecalis* presented by MTA and PC with ZrO_2_ associated with different concentrations of HAn. The null hypothesis is that the addition of HAn would not improve the properties for PC associated with ZrO_2_.

## MATERIAL AND METHODS

**Figure 1:**
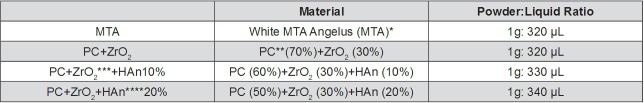
Evaluated materials and powder/liquid proportions

The materials and powder/liquid proportion used are described in Figure 1.

*MTA: Angelus Soluções Odontológicas (Angelus, Londrina, Brazil)

**PC: Portland cement: CP - CPB-40 Estrutural Votoran (Votorantin Cimentos, Camargo Correa Cimentos S.A., Pedro Leopoldo, MG, Brazil)

***ZrO_2_: Zirconium oxide (Sigma Aldrich Corp. St. Louis, MO, USA)

****HAn: Nanoparticulated Hydroxyapatite (HAn - Nanotechnology Laboratory, Physics Institute of São Carlos, SP, Brazil)

### Hydroxyapatite nanoparticle

The HAn was produced using the coprecipitation method. Initially, two aqueous solutions were prepared: a calcium nitrate tetrahydrate solution 1M (Mallinckrodt, 99.9%) and an ammonium phosphate anhydrous 0.6 M solution (Mallinckrodt, 99.4%). The ammonium phosphate solution was dripped into calcium nitrate solution. The pH solution was maintained at approximately 11 with the addition of ammonium hydroxide under N2 flow. Analysis in scanning electron microscopy demonstrated that the obtained particles had a size of 200 nm.

### Setting time

To evaluate the setting time, stainless steel rings with an internal diameter of 10 mm and thickness of 1 mm were filled with the different cements (n=6). The setting time evaluation was performed according to ISO-6876^12^ (2002) and ASTM^7^ (2007) standards, with analyses every 3 minutes during the first half hour, every 5 minutes in the first 2 hours and every 15 minutes from this time forward. To determine the initial setting time, a Gilmore needle with mass of 100±0.5 g and tip diameter of 2±0.1 mm supported on the cement surface was used until the needle could not produce indentations on the cement surface. After determining the initial setting time, measurements continued until the time of the final setting. The specimens were maintained at 37°C during the measurements. To determine the final setting time, the procedures were repeated, using a Gilmore needle with mass of 456±0.5 g and tip diameter of 1±0.1 mm.

### Compressive strength

Specimens (n=6) were fabricated according to BSI-6039^11^ (1981) standards for the compressive strength test. The cements were placed into cylindrical molds with 12 mm of thickness and 6 mm in diameter. They were stored at 37°C and 100% humidity for 3 hours, and then the samples were removed from the mold. The samples remained in the incubator for the period of 24 hours and 21 days. The compressive strength tests were performed in the test machine Emic DL2000, (EMIC Equipamentos e Sistemas de Ensaio Ltda, São José dos Pinhais, PR, Brazil) with 5 kN load cell, at 0.5 mm/minute speed. The maximum stress was recorded in MPa, using the maximum compressive force and the diameter of the cylinders (MPa=N/mm^2^).

### Radiopacity

Radiopacity analysis (n=6) was performed according to ISO-6876^12^ (2002) standards. Samples measuring 10 mm in diameter and 1 mm thickness were manipulated for each material and stored at 37°C and 100% humidity for 48 hours. They were radiographed on an occlusal film (Insight – Kodak Comp, Rochester, NY, USA), next to an aluminum step wedge with a range from 2 to 16 mm thickness. Radiographs were performed using an X-ray device GE 1000 (General Electric, Milwaukee, WI, USA), calibrated at 60 kV, 7 mA, 18 pulses *per* second, and 30 cm of focal distance. The images obtained were digitized and analyzed by the ImageJ software (Fiji 1.46). The areas of the aluminum step wedge and the cements were selected to determine the radiopacity values of the cements in equivalent millimeters of aluminum.

### Solubility

The solubility assay was performed according to ISO-6876^12^ (2002) standards. Sample (n=10) dimensions were 7 mm in diameter and 1 mm thickness, and 5 cm of nylon thread was placed in the cement when the material was inserted into the mold. After 2 and 7 days, the samples were removed from the mold. After removal of residues and loose particles, the samples were placed in a silica desiccator for one hour. The disks and the nylon thread were weighed on a precision scale Model Adventure (Ohaus Corp., Parsuppany, NJ, USA) to obtain the initial mass.

Each of the specimens were held by the thread and put in a plastic container with 10 mL of deionized water, without any contact between the sample and the inner wall of the container. The samples were kept in an oven at 37°C for 15 hours. After this period, the cements were removed from the containers, and washed with distilled and deionized water.

The excess water was removed with absorbent paper. Specimens returned to the silica desiccator for 24 hours, after that they were weighed once again to determine the final mass. Stabilization of each specimen’s weight was confirmed after a new cycle of 24 hours in the silica desiccator. The material solubility corresponded to the mass loss identified for each sample, expressed as a percentage of the original mass.

### pH

The pH analysis (n=10) was performed according to Faria-Junior, et al.^19^ (2013), for which samples measuring 7 mm in diameter and 1 mm thickness were manipulated. Samples were maintained in an oven at 37°C for setting during 2 or 7 days. Then, samples were immersed in plastic flasks with 10 mL of deionized water, with previously determined pH of 6.5. The flasks were closed and kept in an oven at 37°C. The pH measurements were taken with a previously calibrated pH meter, at time intervals of 5 and 15 hours after immersion of the disks. After each measurement, performed in triplicate, the mean pH of each group in each experimental period was calculated.

### Antibiofilm activity

The antimicrobial activity test in direct contact with the biofilm was performed according to Faria-Junior, et al.^19^ (2013). Bovine central incisors were used in the biofilm formation process. The roots were sectioned longitudinally. Dentine blocks measuring 5x5x0.7 mm (width x length x thickness) were obtained using a diamond disc (Isomet – Buheler, Lake Bluff, IL, USA) at low speed, under abundant irrigation. The blocks were put into a test tube containing distilled water for sterilization in an autoclave at 121°C for 20 min.

The microbiological procedures and manipulation of the dentine blocks were performed within a laminar flow chamber (Veco Flow Ltda, Campinas, SP, Brazil). A standard strain of *E. faecalis* (ATCC 29212) was used for biofilm formation. The purity of the strain was confirmed by Gram staining and colony morphology. The microorganisms were reactivated in 4 mL of sterile Brain Heart Infusion broth (BHI, Difco Laboratories Inc., Detroit, MI, USA) and kept in an oven at 37°C for 12 hours. The optical density of the medium was calibrated in a spectrophotometer (Model 600 Plus, Femto, São Paulo, SP, Brazil) set at DO_600_=0.2, which is equivalent to 10^8^ CFU mL^-1^.

The bovine dentine blocks were placed in 24-well culture plates, and each well received 200 µL of the adjusted inoculum and 1.8 mL of BHI medium. The well plates with the submerged bovine dentine block were kept in an incubator (Model Q816M20, Quimis Aparelhos Científicos Ltda., Diadema, SP, Brazil) under constant agitation and microaerophilic conditions at 37°C for 14 days. The samples were kept under constant agitation for uniform biofilm formation on the sample surface, as described by Guerreiro-Tanomaru, et al.^22^ (2013) using confocal laser scanning microscopy. *E. faecalis* is able to develop biofilm on dentin blocks after a period of 14 days^22^. The BHI medium of each well completely changed every 48 hours, without adding new microorganisms, to ensure sufficient nutrients for bacterial growth.

After the post-manipulation periods of 2 and 7 days, according to the method used by Faria-Junior, et al.^19^ (2013), each sample was removed from the mold, sterilized by UV light, and placed over a dentine block containing biofilm, which was previously washed in saline solution to remove the planktonic bacteria. The dentine block/cement sample set was placed in a 48-well plate. On each sample set 20 µL of sterile saline solution was deposited to prevent the dehydration of the sample. The plates were kept at 37°C for the time intervals of 5 and 15 hours of contact. Six discs of each material were used for each period. In the control group Teflon disks with the same size of the cement discs were used. After the contact time, the cement discs were removed and the dentine blocks with remaining biofilm were individually placed in test tubes with 1 mL of sterile saline solution and two glass pearls. The tubes were agitated in vortex (Model Q220, Quimis Aparelhos Científicos Ltda., Diadema, SP, Brazil) for 1 minute to detach the remaining biofilm.

After this, decimal serial dilutions of *E. faecalis* were obtained. Three aliquots of 20 µL of each dilution was inoculated in a Petri dish with *m-Enterecoccus* agar medium (Difco Laboratories – Becton Dickinson and Company - Detroit, MI, USA) and stored in a microaerophilic environment at 37°C for 48 hours.

The readings for each plate resulted in a mean CFU mL^-1^ value in the three areas of bacterial growth that generated between 5 and 50 colonies per field. The number of CFU mL^-1^ was calculated for each cement in each period. The data were submitted to logarithmic transformation to base 10 and the results were presented as the mean of six specimens *per* group.

For all the tests, the results obtained were submitted to a normality test, and with this being proved, they were submitted to the parametric ANOVA statistical test for comparison of the different groups among them and to the Tukey multiple comparison test, with 5% significance.

## RESULTS

### Setting time

Table 1 shows that the MTA group presented the shortest initial setting time in comparison with the other groups (p<0.05), which were similar among them (p>0.05). The shortest final setting time was presented by PC+ZrO_2_+HAn10% group and the longest by MTA group.

**Table 1:** Mean values and standard deviation for initial and final setting time (minutes), compressive strength (MPa) after 24 hours and 21 days and radiopacity (mmAl)

	Setting time	Setting time	Compressive strength	Compressive strength	Radiopacity
	Initial	Final	24 hours	21 days	
MTA	23.67 (± 3.44)^a^	170.7 (± 5.39)^d^	19.63 (± 2.16)^a^	29.51 (± 4.58)^b^	4.52 (± 0.37)^a^
PC+ZrO_2_	42.00 (± 4.05)^b^	151.8 (± 8.97)^c^	23.29 (± 3.23)^a^	45.77 (±2.73)^a^	3.70 (± 0.07)^b^
PC+ZrO_2_+HAn10%	40.33 (± 3.67)^b^	100.5 (± 3.08)^a^	14.41 (± 3.20)^b^	30.90 (± 5.32)^b^	4.18 (± 0.29)^a^
PC+ZrO_2_+HAn20%	40.17 (± 3.06)^b^	119.2 (± 9.28)^b^	4.94 (± 1.48)^c^	19.76 (± 1.94)^c^	3.54 (± 0.20)^b^

Equal letters in the same column indicate statistical similarity (p>0.05)

### Compressive strength

The results obtained point out statistical similarity between MTA and PC+ZrO_2_ groups in the time interval of 24 hours. For the 21 days period, PC+ZrO_2_ group presented the highest value (p<0.05). The lowest value in both experimental times was expressed by PC+ZrO_2_+HAn20% group, as shown in Table 1.

### Radiopacity

PC+ZrO_2_ and PC+ZrO_2_+HAn20% groups presented lower radiopacity when compared with the other groups. MTA and PC+ZrO_2_+HAn10% presented the highest radiopacity values (p<0.05), based on Table 1.

### Solubility

The highest solubility values were shown by PC+ZrO_2_+HAn10% and PC+ZrO_2_+HAn20% groups (p<0.05) in both experimental periods (2 and 7 days). The lowest degree of solubility was presented by MTA and PC+ZrO_2_ groups in 2 and 7 days (Table 2).

**Table 2:** Mean values (%) and standard deviation for solubility and pH evaluation (2- and 7-day setting), after 15 hours of immersion

	Solubility	Solubility	pH	pH
	2 days (%)	7 days (%)	2 days	7 days
MTA	3.70 (± 0.91)^a^	5.54 (± 1.22)^a^	10.99 (±0.07)^b^	10.54 (±0.37)^b^
PC+ZrO_2_	3.49 (± 0.47)^a^	5.97 (± 1.32)^a^	11.3 (±0.10)^a^	10.87 (±0.14)^a^
PC+ZrO_2_+HAn10%	5.96 (± 1.18)^b^	7.91 (± 1.17)^b^	11.3 (±0.11)^a^	11.06 (±0.11)^a^
PC+ZrO_2_+HAn20%	6.45 (± 1.15)^b^	7.63 (± 1.39)^b^	11.38 (±0.06)^a^	10.87 (±0.29)^a^
Control			7.45 (±0.09)^c^	7.29 (±0.20)^c^

Equal letters in the same column indicate statistical similarity (p>0.05)

### pH

There was no difference in pH levels of PC+ZrO_2_, PC+ZrO_2_+HAn (10%) and PC+ZrO_2_+HAn (20%) (p>0.05) and these cements presented higher levels of pH than MTA (p<0.05) as shown in Table 2.

### Antibiofilm activity

In the 2 days experimental period, PC+ZrO_2_+HAn10% and PC+ZrO_2_+HAn20% groups showed the best results in both time intervals, 5 and 15 hours, with PC+ZrO_2_+HAn10% being statistically similar to MTA and PC+ZrO_2_ in the time interval of 5 hours (p>0.05). In the experimental time of 7 days post manipulation, the groups were shown to be statistically similar among them (p>0.05). All the cements presented antibiofilm activity (Table 3).

**Table 3:** Mean values (CFU mL-1) and standard deviation for antibiofilm activity evaluation

	2 days	2 days	7 days	7 days
	5 hours	15 hours	5 hours	15 hours
MTA	6.11 (±0.58)^b^	6.50 (±0.66)^b^	5.75 (±0.15)^a^	5.65 (±0.38)^a^
PC+ZrO_2_	6.39 (±0.36)^b^	6.71 (±0.22)^b^	6.31 (±0.36)^ab^	5.38 (±0.36)^a^
PC+ZrO_2_+HAn10%	5.79 (±0.12)^ab^	4.99 (±0.20)^a^	5.96 (±0.38)^a^	5.35 (±0.43)^a^
PC+ZrO_2_+HAn20%	5.37 (±0.33)^a^	5.44 (±0.26)^a^	6.02 (±0.56)^a^	5.35 (±0.34)^a^
Control	7.11 (±0.35)^c^	7.73 (±0.89)^c^	6.76 (±.53)^b^	6.91 (±0.36)^b^

Equal letters in the same column indicate statistical similarity (p>0.05)

## DISCUSSION

HAn have been associated to materials to enhance their mechanical^2^ and biological^17^ properties. In this study, MTA showed a final setting time of 170 min, in agreement with previous study^10^. The addition of HAn10% and 20% to the PC+ZrO_2_ did not affect the initial setting time and decreased the final setting time. Previous study showed that the addition of HAn reduced the initial setting time of the calcium silicate cement^4^.

The addition of 10% and 20% HAn provided a decrease in the PC+ZrO_2_ compressive strength at 24 hours and 21 days, although the compressive strength of PC+ZrO_2_+HAn10% was similar to MTA at 21 days. This result may have been influenced by the powder/liquid ratio^9^, since in the groups containing Han, a larger amount of water were required to obtain suitable consistency for clinical use. Antonijevic, et al.^4^ (2015) observed that the addition of HAn to calcium silicate cement results in a material with better physicochemical properties without changing its mechanical properties.

The evaluated materials exhibit solubility values greater than the ones established by ANSI/ADA no. 57^3^ (2000), which requires that solubility must be lower than 3%. MTA and PC+ZrO_2_ had lower degree of solubility. PC+ZrO_2_+HAn10% and PC+ZrO_2_+HAn20% presented the highest solubility degree. The hydroxyapatite participates in chemical changes that occur during the initial setting of glass ionomer cement^5^. The reaction of HAn with glass ionomer cement occurs more slowly compared to hydroxyapatite micro size particles, due to the higher crystallinity, lower porosity and lower specific surface area of HAn^6^. This might explain the reduced compressive strength and increased solubility when HAn is added to PC+ZRO_2_.

The addition of 30% ZrO_2_ promotes proper radiopacity for a retrofilling material^16^, according to ISO-6876^12^ (2002) standard. The MTA radiopacity and PC+ZrO_2_+HAn10% was similar and higher than other groups. Moreover, all the cements evaluated presented higher radiopacity than the 3 mmAl recommended for dental cement^12^.

MTA and PC have calcium oxide in their composition, a precursor of calcium hydroxide, responsible for releasing hydroxyl ions and raising the pH^18^. Camilleri, Cutajar and Mallia^13^ (2011) studied the hydration of PC+ZrO_2_ and MTA ProRoot when immersed in HBSS, and showed hydrated calcium, calcium hydroxide and traces of ettringite, relating the hydroxyl ion release and elevation of pH during hydration of the cement. Guerreiro-Tanomaru, et al.^21^ (2012) related the pH value of 10.2 for PC or association to ZrO_2_. There was no difference in pH levels of PC+ZrO_2_, PC+ZrO_2_+HAn (10%) and PC+ZrO_2_+HAn (20%) and these cements presented higher pH than MTA. These results are in agreement with the study which showed that the addition of HAn to calcium silicate cement did not change the pH values compared to Portland cement^4^.

The antimicrobial test by diffusion in agar has been well established in the literature^25^. However, it may be influenced by material solubility and capacity to diffuse into the medium^26^. Also, it is difficult to evaluate materials after setting. The antibacterial activity by counting CFU mL^-1^ is commonly used^19^ ^,^ ^22^, showing the viability of the bacterial strain. The methodology used in this study, described by Faria-Junior, et al.^19^ (2013), allows the evaluation of the antimicrobial property against bacteria in the biofilm form, after the setting reaction of the cements, differently from studies that evaluate the antibacterial action on the planktonic form.

The evaluated materials were not capable of completely reducing *E. faecalis*. The highest level of effect against biofilm was observed for cements containing HAn 10% and 20%, in comparison with MTA and PC+ZrO_2_ after the post-manipulation period of 2 days. The antimicrobial effect of MTA might be due to its high pH or to substances that are released from MTA into the media^27^. An important aspect of hydroxyapatite is the possibility of the substitution of ions with different charges; for example, it is possible to exchange phosphate ions (-3) with carbonate ions (-2)^8^. Such situation leads to the creation of a positively charged vacancy, which is compensated by the release of one cation of calcium (Ca^2+^) and one hydroxyl ion (OH^-^)^8^. Carbonate ions are present in the Portland cement composition^20^. Therefore, it might react with hydroxyapatite and increase the pH, improving the antibacterial activity of MTA-based cements containing HAn. However, there was no increase in the pH of the cements containing HAn in this study.

Hydroxyapatite alone has no antimicrobial activity^23^. However, recent study showed that nanoparticulated hydroxyapatite has bactericidal activity against aerobes, Streptococcus spp. and anaerobes^1^. In this study, the antibacterial activity of PC+ZrO_2_+HAn (10%) and PC+ZrO_2_+HAn (20%) against *E. faecalis* could be attributed to the antibacterial activity of HAn or due to some reaction of HAn with components of PC. However, studies are needed to test these hypotheses.

## CONCLUSIONS

The addition of HAn to PC associated with ZrO_2_ harmed the compressive strength and solubility. On the other hand, HAn did not change the pH and the initial setting time, but improved the radiopacity (HAn 10%), the final setting time and the *E. faecalis* antibiofilm activity of the cement.

## References

[1] Abdulkareem EH, Memarzadeh K, Allaker RP, Huang J, Pratten J, Spratt D (2015). Anti-biofilm activity of zinc oxide and hydroxyapatite nanoparticles as dental implant coating materials. J Dent.

[2] Arcís RW, López-Macipe A, Toledano M, Osorio E, Rodríguez-Clemente R, Murtra J (2002). Mechanical properties of visible light-cured resins reinforced with hydroxyapatite for dental restoration. Dent Mater.

[3] American National Standards Institute, American Dental Association (2000). Specification no. 57: endodontic sealing materials.

[4] Antonijevic D, Jeschke A, Colovic B, Milovanovic P, Jevremovic D, Kisic D (2015). Addition of a fluoride-containing radiopacifier improves micromechanical and biological characteristics of modified calcium silicate cements. J Endod.

[5] Arita K, Lucas ME, Nishino M (2003). The effect of adding hydroxyapatite on the flexural strength of glass ionomer cement. Dent Mater J.

[6] Arita K, Yamamoto A, Shinonaga Y, Harada K, Abe Y, Nakagawa K (2011). Hydroxyapatite particle characteristics influence the enhancement of the mechanical and chemical properties of conventional restorative glass ionomer cement. Dent Mater J.

[7] American Society for Testing Materials (2007). International C266-07. Standard test method for time of setting of hydraulic cement paste by Gillmore needles.

[8] Barralet J, Best S, Bonfield W (1998). Carbonate substitution in precipitated hydroxyapatite: an investigation into the effects of reaction temperature and bicarbonate ion concentration. J Biomed Mater Res.

[9] Basturk FB, Nekoofar MH, Günday M, Dummer PM (2013). The effect of various mixing and placement techniques on the compressive strength of mineral trioxide aggregate. J Endod.

[10] Bosso-Martelo R, Guerreiro-Tanomaru JM, Viapiana R, Berbert FL, Duarte MA, Tanomaru M (2016). Physicochemical properties of calcium silicate cements associated with microparticulate and nanoparticulate radiopacifiers. Clin Oral Investig.

[11] British Standards Institution (1981). BS 6039:1981 - Specification for dental glass ionomer cements.

[12] British Standards Institution (2002). BS EN ISO 6876:2002. Dental root canal sealing materials.

[13] Camilleri J, Cutajar A, Mallia B (2011). Hydration characteristics of zirconium oxide replaced Portland cement for use as a root-end filling material. Dent Mater.

[14] Collares FM, Leitune VC, Rostirolla FV, Trommer RM, Bergmann CP, Samuel SM (2012). Nanostructured hydroxyapatite as filler for methacrylate-based root canal sealers. Int Endod J.

[15] Cornélio AL, Rodrigues EM, Salles LP, Mestieri LB, Faria G, Guerreiro-Tanomaru JM (2015). Bioactivity of MTA Plus, Biodentine and experimental calcium silicate-based cements in human osteoblast-like cells. Int Endod J.

[16] Cutajar A, Mallia B, Abela S, Camilleri J (2011). Replacement of radiopacifier in mineral trioxide aggregate; characterization and determination of physical properties. Dent Mater.

[17] Dasgupta S, Tarafder S, Bandyopadhyay A, Bose S (2013). Effect of grain size on mechanical, surface and biological properties of microwave sintered hydroxyapatite. Mater Sci Eng C Mater Biol Appl.

[18] Duarte MA, Demarchi AC, Yamashita JC, Kuga MC, Fraga SC (2003). pH and calcium ion release of 2 root-end filling materials. Oral Surg Oral Med Oral Pathol Oral Radiol.

[19] Faria NB, Tanomaru M, Berbert FL, Guerreiro-Tanomaru JM (2013). Antibiofilm activity, pH and solubility of endodontic sealers. Int Endod J.

[20] Garrabrants AC, Sanchez F, Kosson DS (2004). Changes in constituent equilibrium leaching and pore water characteristics of a Portland cement mortar as a result of carbonation. Waste Manag.

[21] Guerreiro-Tanomaru JM, Cornélio AL, Andolfatto C, Salles LP, Tanomaru M (2012). pH and antimicrobial activity of Portland cement associated with different radiopacifying agents. ISRN Dent.

[22] Guerreiro-Tanomaru JM, Faria NB, Duarte MA, Ordinola-Zapata R, Graeff MS, Tanomaru M (2013). Comparative analysis of Enterococcus faecalis biofilm formation on different substrates. J Endod.

[23] Lim PN, Teo EY, Ho B, Tay BY, Thian ES (2013). Effect of silver content on the antibacterial and bioactive properties of silver-substituted hydroxyapatite. J Biomed Mater Res A.

[24] Mestieri LB, Tanomaru M, Gomes-Cornélio AL, Salles LP, Bernardi MI, Guerreiro-Tanomaru JM (2014). Radiopacity and cytotoxicity of Portland cement associated with niobium oxide micro and nanoparticles. J Appl Oral Sci.

[25] Morgental RD, Vier-Pelisser FV, Oliveira SD, Antunes FC, Cogo DM, Kopper PM (2011). Antibacterial activity of two MTA-based root canal sealers. Int Endod J.

[26] Nawal RR, Parande M, Sehgal R, Naik A, Rao NR (2011). A comparative evaluation of antimicrobial efficacy and flow properties for Epiphany, Guttaflow and AH-Plus sealer. Int Endod J.

[27] Parirokh M, Torabinejad M (2010). Mineral trioxide aggregate: a comprehensive literature review - Part I: chemical, physical, and antibacterial properties. J Endod.

[28] Suzuki M, Taira Y, Kato C, Shinkai K, Katoh Y (2015). Histological evaluation of direct pulp capping of rat pulp with experimentally developed low-viscosity adhesives containing reparative dentin-promoting agents. J Dent.

[29] Venkatesan J, Kim SK (2014). Nano-hydroxyapatite composite biomaterials for bone tissue engineering - a review. J Biomed Nanotechnol.

[30] Wu BC, Wei CK, Hsueh NS, Ding SJ (2015). Comparative cell attachment, cytotoxicity and antibacterial activity of radiopaque dicalcium silicate cement and white-coloured mineral trioxide aggregate. Int Endod J.

